# A Dirichlet‐Multinomial Gibbs Algorithm for Assessing the Accuracy of Binary Tests in the Absence of a Gold Standard

**DOI:** 10.1002/sim.70372

**Published:** 2026-01-22

**Authors:** Joseph B. Kadane

**Affiliations:** ^1^ Department of Statistics & Data Science Carnegie Mellon University Pittsburgh Pennsylvania USA

**Keywords:** *chlamydia*, Dirichlet‐multinomial distribution, Gibbs sampler, Markov chain Monte Carlo, sensitivity, specificity

## Abstract

Each patient is simultaneously given several binary tests for a disease. The tests are partitioned into disjoint groups, assumed to be conditionally independent between groups, but allowed to have arbitrary dependence within a group. The groups are intended to capture similar biological features of the tests. A Dirichlet‐multinomial model is employed with a Gibbs Sampler to estimate the sensitivity and specificity of the tests. The model is exemplified by data on four tests for Chlamydia, both with complete data and with a random 10% of the data treated as missing.

## 1 Part I: Complete Data

### 1.1 Introduction

The data in this problem are the outcome of each test performed on each patient. The goal is to approximate posterior distributions for the sensitivity (the probability that a person with the disease will test positive) and the specificity (the probability that a person without the disease will test negative) for each test. In this part, we assume that each patient is given each test. Parts 3 and 4 address the case in which some of the data are missing.

Suppose there are n patients, each of whom is given k tests. Then there are kn data points and n+2k parameters specifying the latent disease status for each patient, and the sensitivity and specificity for each test. When k=1, there are more parameters than observations, leading to a situation in which informative priors must be imposed, and to which the target parameters, the sensitivity and specificity, would then be sensitive. Consequently, the recommendation is that k be at least two.

An additional feature of the problem is that there is a label switching issue. If one believes that the tests are generally good, then positive test results are associated with a greater likelihood of positive disease status and conversely. If one believes that all the tests are generally catastrophically poor, then positive test results are associated with a greater likelihood of negative disease status, again conversely. The model is unidentified, as the data equally support both views, as is shown below.

There is a large literature on the use of multiple tests in the absence of a gold standard, mainly with medical and veterinary applications. For reviews see [1]; [2] and [3]. Reference [4] focus on conditionally independent tests in at least two populations with different prevalences, using maximum likelihood estimation. Reference [5] analyzed two conditionally independent tests from a single population using a Bayesian method with informative priors. Reference [6] also discussed Bayesian approaches to diagnostic outcome data, with two conditionally independent tests. They highlighted the potential lack of consistency of estimators due to the lack of identifiability in the one‐population case and also discussed the fully Bayesian approach to the two‐test two‐population version of the Hui and Walter model. Reference [7] model two dependent tests with covariance parameters, using Bayesian methods to mitigate the lack of identifiability of the two‐test one‐population model, and [8] developed an alternative dependence model for this type of data. Their paper specifically addresses the issue of the effect of correlation on their Bayesian inferences, which is shown to be minimal if the correlation between tests is less than 0.20, and substantial if it is greater than 0.4. Reference [9] review several Bayesian models for multiple tests, using veterinary examples. Reference [10] extended the Dendukuri and Joseph model to multiple tests, and provided methods for assessing the identifiability of diagnostic testing models. Reference [11] showed the potential lack of robustness of some frequentist dependence models.

The remainder of this paper is organized as follows: Section 1.2 explains why this is a difficult problem, and why an iterative sampler is a natural solution. The proposed algorithm is introduced and explained. Part 2 applies the algorithm to a data set of patients tested for chlamydia using four tests, two of which are believed to be correlated. Part 3 discusses modifying the analysis to deal with missing data. Part 4 implements the material in Part 3 to the chlamydia data, with randomly chosen 10% of the data missing.

### 1.2 The Problem and the Algorithm

#### 1.2.1 Why Estimation of Sensitivity and Specificity is a Hard Problem With Only One Test Per Patient

Suppose $m_{p}$ patients test positive and $m_{n}$ test negative. Let $\theta_{p}$ be the probability that someone who tests positive is diseased, and let $\theta_{n}$ be the probability that someone who tests negative has the disease. Finally, let ψ be the prevalence of the disease in the population. Then ψ can be estimated as

(1) $$\begin{array}{ll} \psi & =P\{D=1|\text{testpos}\}P\{\text{test pos}\}+P\{D=1|\text{testneg}\}P\{\text{test neg}\}\\ & =(\theta_{p}m_{p}+\theta_{n}m_{n})/(m_{p}+m_{n}), \end{array}$$

where D=1 if the patient is diseased and 0 otherwise.

Suppose that the test has sensitivity se and specificity sp. If there were a “gold standard,” a sure way to determine who has or does not have the disease, it would be simple to estimate the sensitivity and specificity of the test. However, without a gold standard, one must rely on the tests in question to determine a patient's disease status.

Conversely, suppose that se and sp were known. Then Bayes theorem applies, and yields

(2) $$\theta_{p}=P\{D=1|\;\text{positive test}\}=\psi se/(\psi se+(1-\psi )sp),$$

called the positive predictive value (PPV).

Similarly

(3) $$\begin{array}{ll} \theta_{n} & =P\{D=1|\;\text{negative test}\}\\ & =\psi (1-se)/(\psi (1-se)+(1-\psi )sp). \end{array}$$

This is one minus the negative predictive value (NPV).

Let $X_{i}=1$ if person i tests positive, and 0 otherwise. Then (2) and (3) can be written jointly as

(4) $$P\{D=1|X_{i}\}=\frac{\psi se^{X_{i}}{\left(1-se\right)}^{1-X_{i}}}{\psi se^{X_{i}}{\left(1-se\right)}^{1-X_{i}}+(1-\psi )sp^{1-X_{i}}{\left(1-sp\right)}^{X_{i}}}.$$

Thus, if one knows the sensitivity and specificity of the test and has an estimate of ψ, the probability that a particular person has the disease, given their test outcome, is available. And, if one knew the disease status of each tested patient, the sensitivity and specificity of each test and $\theta_{t}$ could be estimated. The fact that neither source of information is available is what makes this a challenging problem.

Sensitivity and specificity are simple to estimate if the disease status were known. Similarly, if sensitivities and specificities were known, Bayes theorem in (4) offers a route to disease status. It is natural, then, to consider an algorithm that iterates between these.

### 1.3 Notation and a Sketch of the Algorithm

With several tests, the notation needs to be expanded. Patients are now distinguished by their outcomes on all the tests. A patient's “type” t specifies those outcomes on each test. See Table 1 to come for an example. Suppose that there are $m_{t}\geq 0$ patients of type t, for t=1,…,T and let $m=(m_{t},\ldots ,m_{T})$ and let $\theta =(\theta_{t},\ldots ,\theta_{T})$. Each patient type is characterized by $\theta_{t}$, the probability that a patient of type t has the disease. In Section 1.1, the patient types were “p” and “n,” positive and negative test results on the single test discussed there.

**TABLE 1:** Number of patients of each type, and their frequency.

Type	LCR	PCR	DNAP	Culture	Obs	Frequency in percent
1	1	1	1	1	210	5.9
2	1	1	1	0	12	0.3
3	1	1	0	1	44	1.2
4	1	1	0	0	59	1.7
5	1	0	1	1	32	0.9
6	1	0	1	0	0	0
7	1	0	0	1	10	0.3
8	1	0	0	0	39	1.1
9	0	1	1	1	14	0.4
10	0	1	1	0	0	0
11	0	1	0	1	8	0.2
12	0	1	0	0	27	2.7
13	0	0	1	1	18	0.5
14	0	0	1	0	11	0.3
15	0	0	0	1	24	0.7
16	0	0	0	0	3043	85.7

The remainder of this section offers an outline of the steps in the proposed algorithm, details of which are explained in sections that follow. A single trip through these steps is called an iteration, indexed by c. The steps of the algorithm are:

A. Given $\theta =(\theta_{1},\ldots ,\theta_{T})$, simulate disease presence $N=(N_{1},\ldots ,N_{T})$, where $N_{1}$ is the number of patients of type t who are diseased. (Section 1.4)
B. Given the result of N, simulate sensitivity and specificity of each test.
  a. Assuming independence among the tests (Section 1.5)
  b. Allowing for dependence among the tests (Section 1.6)
C. Calculate the prevalence ψ. (Section 1.7)
D. Using ψ and the sensitivities and specificities simulated in step B, apply Bayes theorem to calculate new probabilities θ. (Section 1.8)
E. Return to A.

### 1.4 Step A: Simulation of Disease Presence

For each type t, the data give the number, $m_{t}$, of patients of that type. Also, $\theta_{t}$ is the probability that a patient of type t has the disease. It is then natural to endow $N_{t,c}$, the number of patients of type t who have the disease (for iteration c), to have a Binomial $\left(m_{t},\theta_{t}\right)$ distribution.

### 1.5 Step Ba: Sensitivity and Specificity, Given Disease Status, Independence Case

To be given the disease status of each individual (temporarily, for this iteration) means that it is “known” which individuals have the disease and which do not. Only those with the disease are relevant to the sensitivity of each test. Similarly, only those without the disease are relevant to the specificity of each test. Thus, the data on the relevant individuals indicate for each test whether the test accurately predicted disease status positive (sensitivity) or negative (specificity). Because of this symmetry, it is necessary to deal only with updating sensitivity among those who have the disease (for this iteration). Specificity among those who do not have the disease (for this iteration) is analogous.

The simplest model to consider is to suppose that the sensitivities (specificities) of tests are conditionally independent given disease states. In this case, the data are Bernoulli. Let $s_{j}$ be the sensitivity (specificity) of the $j^{th}$ test. Suppose $C_{j}$ individuals were correctly predicted by test j, and $E_{j}$ individuals were not. Then the likelihood contribution is proportional to

(5) $$s_{j}^{C_{j}}{\left(1-s_{j}\right)}^{E_{j}.}$$

With the specified independence assumption, the marginal distribution of the Dirichlet distribution is beta distributions.

If a beta $\left(\alpha_{j},\beta_{j}\right)$ prior is imposed on the sensitivity (specificity) of test j, then the posterior distribution is beta ($\left(\alpha_{j}+C_{j},\beta_{j}+E_{j}\right)$). A random draw from this posterior distribution specifies the sensitivity (specificity) to be passed to the other steps of the algorithm.

### 1.6 Step Bb: Possibly Dependent Tests

There's every reason to think that tests that are based on the same biological phenomena might be stochastically dependent [12]. This would mean, for instance, that a false negative on one test might raise the probability of a false negative on another closely related test. The implication of possibly dependent tests is that new modeling is required, and that the same data set modeled taking dependence into account will have less information about test characteristics than would an independence model.

The context of groups of tests requires revisiting what is meant by “sensitivity” and “specificity.” These terms might refer to the vector of sensitivities and specificities, or they might refer to only the marginals of these vectors. For the purpose of advancing the algorithm, the former is what is required. However, for reporting the outcome of the analysis, the question comes down to what decision problem the results will be used to inform. If the idea is to choose one test to be used routinely, then results on the sensitivity and specificity of each test individually is presumably the relevant report. However, if the idea is to choose a set of tests (i.e., a member of the power set of tests) to recommend, then the relevant report will specify the sensitivity and specificity of each possible outcome on each test in each candidate subset of tests. A full decision‐theoretic treatment would take into account the cost of each choice, in terms of money and hassle (for patients and the medical personnel). It should also specify the recommended course of action as a function of test results. The latter is especially important when the choice is to give several tests whose findings may differ. What alternative subsets of the power set of tests to explore should be decided separately in each application?

Suppose that the tests are divided into groups, where it is acceptable to assume independence between groups, but not within groups. To keep the terminology clear, “type” refers to a set of individuals whose outcomes on all the tests are identical. “Group” refers to a set of tests. Test outcomes are assumed to be independent between groups, but not necessarily within a group. If each group is of size one, then the problem is reduced to the independence case already discussed. Hence, attention is focused on a group of tests, assumed to be possibly dependent. It suffices to state the model for one such group, consisting of g>1 tests.

The result of such tests on an individual can be thought of as a g‐dimensional vector of ones and zeros, one for a positive test and zero for a negative test. The space of results has $2^{g}$ elements. At each iteration of the sampler, there will be two tables of length $2^{g}$, one for those with positive imputed disease status, and one for those with negative imputed disease status. A Dirichlet distribution with parameter α on this space can be updated with the data, which changes from iteration to iteration because the imputation of disease status changes as iterations proceed. Thus, corresponding to each of the tables is a vector of length $2^{g}$ of updated hyperparameters α, reflecting the prior (which does not change from iteration to iteration) and the “data,” which does.

Within a group, the Dirichlet model proposed here is unrestrictive in that it can fit arbitrary dependence since it is applied to the power set of the tests in the group. This possibly can be a strength. It can also be a weakness, as it introduces $2^{g}$ parameters each for sensitivity and specificity.

The flexibility of assuming a Dirichlet distribution on the power set contrasts with other approaches that depend on correlations and covariances. While it is reasonable to suppose that dependence among tests' sensitivities and specificities will generally be positive, it seems wise to allow for the possibility of negative dependence. The following result in [13] shows a limitation of measuring multivariate dependence with correlation:

Let $X_{i},i=1,\ldots ,n$ have arbitrary means and variances. Let $\rho (X_{i},X_{j})=\rho_{i,j}$. Then

(6) $$\bar{\rho }=\sum_{i\neq j}\rho_{ij}/n(n-1)\geq -1/(n-1).$$

Because beta distributions are used to model sensitivity and specificity in the independence case, it is natural to look to dependent extensions of the beta distributions to model possibly dependent sensitivities and specificities. A review of such bivariate extensions is given by [14]. They criticize the use of a Dirichlet model because “it is restricted to a lower dimensional simplex.” Presumably, Olkin and Trikalinos mean by this the g−1 dimensional simplex. By contrast, the Dirichlet model proposed here is on the $2^{g}-1$ dimensional simplex on the power set of the elements of the group.

When and whether to assume conditional independence of tests, and the resulting quality of estimates of sensitivity and specificity, has engaged scholars for many years. References [15] and [16] pointed to this issue. Vasek wrote “Often this assumption of conditional independence cannot be justified, particularly if the tests are based on the same physiologic phenomenon. For example, if both tests are based on a particular antibody reaction, something which inhibits the reaction or causes a fake reaction for one of the tests may have a similar effect on the other.” Some of the ensuing studies can be found in [7, 11, 17, 18, 19, 20, 21, 22] and [23] and the references therein.

The current study picks up from Vasek's thought quoted above, and groups tests based on their biology. Thus, tests with similar “physiologic phenomena” would be grouped together, independent of tests based on distinct biology. In this view, tests are not treated as black boxes regarded symmetrically, but rather treated according to the biological aspects each presents. However, many studies showing how a casual assumption of conditional independence of tests leads to misleading results is a good warning that assumptions about how the tests relate to each other are critical. How seriously the resulting sensitivity and specificity estimates should be taken depends on how satisfied one is with the assumptions used in deriving them.

### 1.7 Step C: Calculate Prevalence ψ

Generalizing Equation (1) to reflect T types of patients,

(7) $$\psi =\overset{T}{\underset{t=1}{\sum }}\theta_{t}m_{t}/\overset{T}{\underset{i=1}{\sum }}m_{t}$$

where ψ is the prevalence, the (unconditional) probability that a patient is diseased. It is important to note that (7) is conditionally deterministic, in that it does not require an additional drawing from a distribution. Patients may be regarded as belonging to different populations that may differ in prevalence. In that case, Equation (7) can be used separately in each population.

### 1.8 Step D: Disease Probability, Given Type Probabilities, Data, Sensitivities and Specificities

This section specifies how to simulate disease probability for each individual i of type t. This is accomplished by a generalization of (4) that takes account of all the data.

Suppose there are K groups of tests, indexed by k. A given type t of patient specifies the outcome $z_{k}$ of all tests in group k, for k=1,…,K. Then

(8) $$\begin{array}{ll} \theta_{t} & =P\{D=0|t,\psi \}\\ & =\frac{\psi \overset{K}{\underset{k=1}{\prod }}P\{z_{k}|D=0\}}{\psi \left[\overset{K}{\underset{k=1}{\prod }}p\{z_{k}|D=0\}\right]+(1-\psi )\left[\overset{K}{\underset{k=1}{\prod }}p(z_{k}|D=0)\right]}. \end{array}$$

These are the new disease probabilities for each patient of type t, returned to step A in the outline of Section 1.4. Note that (8) is again conditionally deterministic in character.

If there are separate population prevalences, then the concept of “type” of patient includes both the outcome of all tests and the specification of the prevalence population to which he or she belongs. Then the quantity in (8) still applies, using the relevant population prevalence.

### 1.9 Characteristics of the Algorithm

This algorithm is Bayesian, which means that the data are regarded as fixed at their observed values, while the parameters, being uncertain quantities, have distributions. In the alternative frequentist framework, parameters are regarded as fixed but unknown, and the data, even after they are observed, are regarded as random.

Identification of parameters is often regarded as an issue in the literature. This is a concern in frequentist statistics, but not necessarily for Bayesians. A Bayesian with a proper prior and a likelihood can compute a posterior distribution on the parameters, regardless of the identifiability of the likelihood. To give an example, suppose $X_{i}∼N(\mu_{i},1)i=1,2$, where $X_{1}$ and $X_{2}$ are independent. Suppose that the data are observations on $X_{1}+X_{2}$. Then $\mu_{1}$ and $\mu_{2}$ are not identified, because observations from $N(\mu_{1}+c,1)$ and $N(\mu_{2}-c,1)$ are indistinguishable from observations on $N(\mu_{1},1)$ and $N(\mu_{2},1)$. With a proper prior in $\left(\mu_{1},\mu_{2}\right)$ space, the posterior distribution will change under the specified likelihood, so the Bayesian has learned from the data, even without identifiability.

The vector θ is the only information passed from one iteration to the next. Since no past values of θ are relevant, the process is Markovian, and the values of α,N,se, and sp form a Markov Chain. Additionally, it is a Monte Carlo algorithm because it relies on random draws from distributions.

Every statement in the following paragraph is conditional on θ. The distribution of N is Binomial and does not depend on se or sp. The distribution of se(sp) depends on N. Thus, the first is an unconditional marginal distribution, and the second is conditional. Their product defines a joint probability distribution on N,se and sp.

Furthermore, steps A and B use conjugate distributions, so the algorithm is a Gibbs sampler, a special case of the Metropolis Hastings algorithm. Under very general conditions, such algorithms converge to a stationary distribution regardless of the starting condition [24]. In principle, a Gibbs sampler (like all Markov chain Monte Carlo samplers) visits all possible values of the parameters, here N,se and sp.

Standard practice in analyzing the results of a Metropolis Hasting algorithm recommends excluding the starting iterations of the chain as “burn‐in,” and possibly thinning the rest to reduce the autocorrelation between results [25]. CODA [26] is a program that offers several options to guide these decisions.

## 2 Part II: Complete Data, Chlamydia

### 2.1 Introduction

*Chlamydia trachomatis* is a sexually transmitted bacterial infection that is often asymptomatic. However, it can have serious consequences, such as infertility.

The data studied here are from [27] and are given in Table 1. They compare the results of four tests for chlamydia. The first two, ligase chain reaction (LCR) and polymerase chain reaction (PCR), are nuclear acid amplification tests aimed at detecting the DNA of chlamydia. According to [27] “both are believed to be highly sensitive,” but not necessarily very specific. Since LCR and PCR are aimed at the same biological mechanism, they are believed to be possibly correlated. A third test, DNAP, is a DNA probe, thought to have high specificity but lower sensitivity than LCR and PCR. Finally, a fourth test, called the culture test, is thought to have high specificity but sub‐optimal sensitivity. For more on these tests see [27] and [28].

The raw data, given in Table 1, shows 16 types of patients; within a type, all patients have the same test outcomes. Note that every patient was given all four tests, so that there are no missing data.

To model these data, LCR and PCR are regarded as belonging to a group, conditionally independent of DNAP and Culture, which are also assumed to be conditionally independent of each other. In this, I follow [27] who also regard LCR and PCR as related. As explained above, this assumption is critical in the sense that other choices could lead to rather different results. This analysis also uses independent uniform prior distributions for the beta and Dirichlet distributions. This choice is hypothesized to be much less important than the grouping choice.

The main parameters of interest are the sensitivity and specificity of the four tests. Thus, we choose not to calculate and report the sensitivity and specificity of larger sets of tests. The prevalence is a function of the specific population in the data set, so there is nothing to be learned about the tests from the prevalence parameter. (The Gibbs sampler produces a prevalence value each iteration; it is just not interesting for those concerned about properties of the tests).

### 2.2 Label Switching

Label switching is a kind of nonidentification, originally associated [29] with mixture models of the type

(9) $$f_{\xi }(x)=\overset{k}{\underset{j=1}{\sum }}p_{j}f(x|\xi_{j})$$

where $\xi =(\xi_{1},\ldots ,\xi_{k},p\ldots ,p_{k})$ and $f(x|\xi_{j})$ is a specified parametric family. This likelihood is invariant under permutation of the indices. Some of the issues this causes, particularly to Bayesian Markov chain Monte Carlo algorithms, are discussed, for example, in [30] and [31].

An analogous problem occurs in the Gibbs sampler described above. The model admits what I call a mirror solution, in which a positive test result indicates that the person does not have the disease in question, and conversely, a negative test result indicates that the person does have the disease. This changes the meaning of the parameter (N,se,andsp) as follows:

$$N^{*}=m-N,{\mathrm{Se}}^{*}=1-\mathrm{Se},\;\text{and}\;{\mathrm{Sp}}^{*}=1-\mathrm{Sp}.$$

$$\begin{array}{ll} Let & h(N,\mathrm{Se},\mathrm{Sp}),=(m-N,1-\mathrm{Se},1-\mathrm{Sp}),\;\text{and note that}\\ & h(h(N,\mathrm{Se},\mathrm{Sp}))=(N,\mathrm{Se},\mathrm{Sp}). \end{array}$$

When a Markov chain Monte Carlo chain is run on a mixture model, the result (if the chain visits the whole parameter space) is a multimodal distribution difficult to describe (see especially [30]). With the model studied in this paper, the result would be bimodal, but a similar awkwardness would occur.

With respect to the chlamydia data, the likelihood (probability of the data) at (N,Se,Sp) is the same as that at h(N,Se,Sp). The prior does not distinguish between these two points either, so suppose we treat the points (N,Se,Sp) and h(N,Se,Sp) as a single unit, with the probability associated with either (does not matter which since the probabilities are identical). Thus, a visit of the chain to either is a visit to both. This reparameterization eliminates label switching as a source of nonidentification. Whether the resulting parameterization is identified is an open question.

The hyper priors used in the model are uniform for both the beta distributions and the Dirichlet distributions. As a result, each parameter (N,Se,Sp) and its mirror image $\left(N^{*},Se^{*},Sp^{*}\right)$ have the same probability. Hence, the probability of the set $\left\{(N,Se,Sp)(N^{*},Se^{*},Sp^{*})\right\}$ is twice the probability of each component. This factor of 2 is irrelevant to an MCMC, as it moves in a space proportional to the posterior distribution. Hence, for many purposes, the decision to work in the reduced space that associates a parameter point and its mirror point is not relevant to the functioning of the algorithm.

Another thought is that Bayesians can ignore the issue of identifiability. Both the model and a proper prior are available, so a Bayesian can compute the posterior distribution of the parameters (a.k.a. unknown quantities) without reference to identification.

### 2.3 The Algorithm Implemented

Table 1 shows that over 85% of the patients in this study tested negative on all four tests, and nearly 6% tested positive on all the tests. Thus, the information on what distinguishes the sensitivity and specificity of the tests depends on the remaining 9% of the data.

There's an aspect of the choice of starting values that deserves discussion. The conclusion of Section 2.2 above (on label switching) suggests that a parameter vector and its h‐transforms have the same probability for the data, and hence might be regarded as equivalent. Such a perspective changes the topology and geometry within which the chain operates. How to think about Markov chain Monte Carlo methods when the objects are sets of parameters whose constituents are distant in the usual metrics is left here as an unresolved issue.

Finally, as part of the prior specification, the label switching issue has to be addressed. As mentioned several times, a parameter vector and its h‐transform have the same probability for the data. Hence, what distinguishes them is purely a subjective prior matter. Suppose that the estimated sensitivities and specificities of the tests came out to be roughly 80% or equivalently 20%. How much weight would I place on each? In this application, I would believe 80% and not 20%. This choice violates [32], p. 129‐131 “Cromwell Rule,” not to put zero probability on anything possible, but that is how I understand this applied problem.

I report on a run of 100 000 with a burn‐in of 300, no thinning, and a starting vector θ of length 16 with 0.5 for each entry. The first question to address is whether the chain has converged. One way to get evidence on this question is to use Geweke's test [33], which asks whether the first 10% has a different mean than the last 50%, taking account of the dependence structure in the chain. Table 2 records the results:

**TABLE 2:** Geweke tests of mean equality of the first 10% and the last 50%.

DNAP	Culture	LCR	PCR
Sensitivity			
0.0188	0.5190	−0.8266	−0.8281
Specificity			
−0.2105	03661	−1.6516	−04483

Since all of these tests give Geweke numbers substantially less than 2 in absolute value, we may take it as evidence of convergence.

**FIGURE 1:**
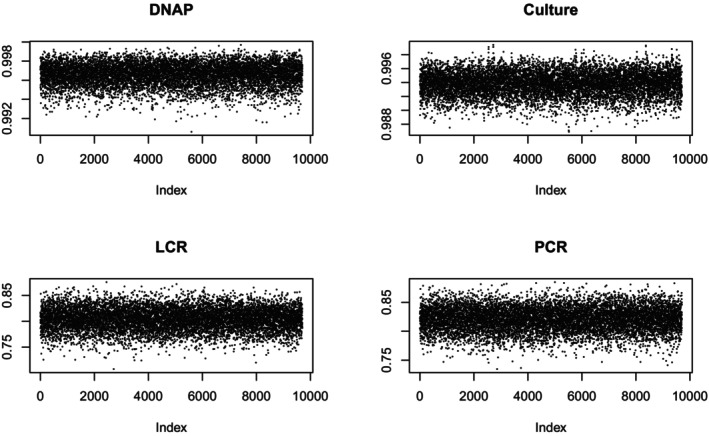
Specificity Trace Plots.

**FIGURE 2:**
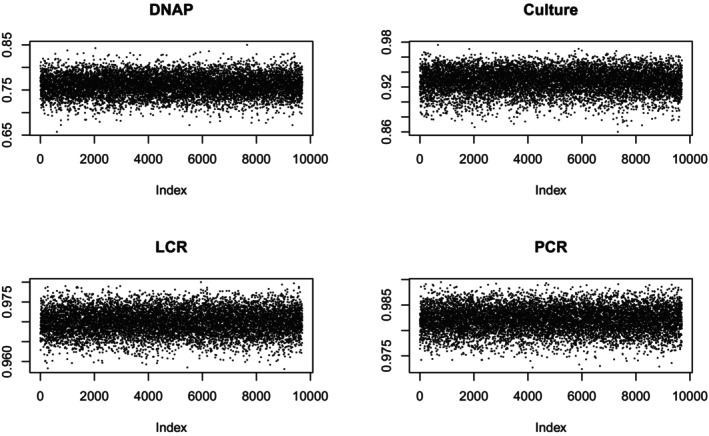
Sensitivity Trace Plots.

A second way to check for convergence is with a trace plot, which shows the data against the index of the run. The point of looking at a trace plot is to look for evidence of a trend. Figures 1 and 2 report trace plots for sensitivity and specificity, respectively. They show no obvious trends. Hence, I feel comfortable proceeding to examine the results. Table 3 shows the basic results. The Appendix gives more detail.

**TABLE 3:** Comparison of results of this paper with those of [27], median and 95% credible interval.

			Results of this paper	Results of MLVM‐RE model [27]
Specificity	DNAP	0.9966	(0.9940, 0.9983)	0.997 (0.994, 0.998)
	Culture	0.9933	(0.9897, 0.9962)	0.994 (0.990, 0.997)
	LCR	0.9694	(0.9631, 0.9752)	0.969 (0.963, 0.975)
	PCR	0.9731	(0.9670, 0.9785)	0.973 (0.967, 0.978)
				
Sensitivity	DNAP	0.8055	(0.7596, 0.8475)	0.798 (0.753, 0.843)
	Culture	0.9538	(0.9233, 0.9748)	0.952 (0.921, 0.973)
	LCR	0.8688	(0.8282, 0.9032)	0.859 (0.817, 0.897)
	PCR	0.8127	(0.7673, 0.8524)	0.803 (0.757, 0.844)

Not only the data, but also the fundamental insight about the relationship of the tests come from Dendurkuri et al. They give reasons why the LCR and PCR tests might be related, as both are nucleic‐acid amplification tests, and hence might be positively correlated, conditional on the disease status of the patient. Their model allows for a DNA latent variable relating these two tests, in a multiple latent variable model MLVM and in the preferred version, and adds a random effect as well (MLVM‐RE).

Table 3 compares the results of these two models. Both the medians and the 95% credible intervals are remarkably close.

All four tests have median specificities in the high 90%'s. Additionally, the Culture test is substantially more sensitive than the other three tests. Thus, LCR and PCR are not highly sensitive, contrary to expectation, but they are quite specific. As expected, DNAP has lower sensitivity than LCR, but (unexpectedly) not PCR. Hence, some, but not all, of the background expectations are supported.

That the specificities have tighter credible intervals than do the sensitivities is likely due to the fact that the preponderance of the patients in this data set do not have chlamydia.

**FIGURE 3:**
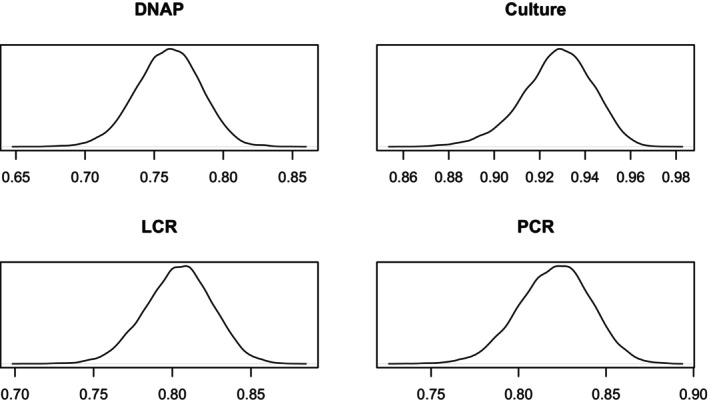
Sensitivity Density Plots.

**FIGURE 4:**
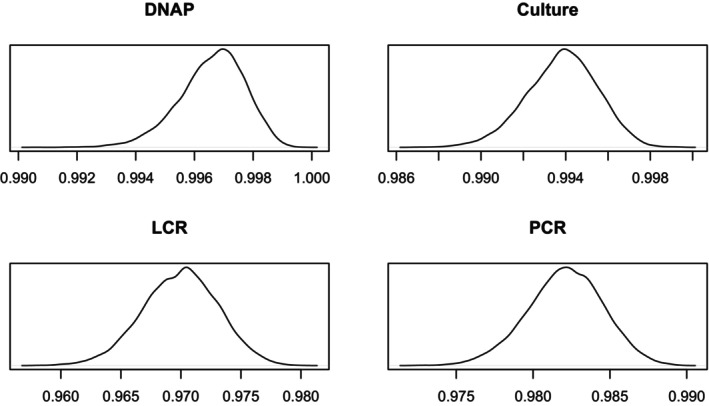
Specificity Density Plots.

Figures 3 and 4 show the densities of the sensitivities and specificities. More details are given in Table A1 in the Appendix.

### 2.4 Discussion of Parts I and II

Table 3 shows that the results found here and those of Dendukuri et al. are very close, which can be taken as confirming both. As a “proof of concept,” this similarity is useful. As to testing for chlamydia, the results suggest that the Culture test is substantially more sensitive than the other tests, and almost as specific.

Methodologically, I would point to the construction of a completely flexible representation of conditional dependence within a group of tests. Additionally, some of the material on the mirror effect shown in Part 1 Section 1.2, is new.

This research effort began during COVID, as a reaction to the fact that there were over 300 tests given Emergency Use Authorization, without knowledge of in vivo sensitivity and specificity. While that emergency is behind us, it would be foolish not to learn what we can from that emergency before the next one happens.

## 3 Part III: Incomplete Data

Bayesian theory recognizes only two kinds of variables: those known (otherwise called data) and those about which there is uncertainty (otherwise called parameters). When data are missing, there is uncertainty about what the value would have been (counterfactually) had it been observed. Consequently missing data are simply another kind of parameter. Since in the Bayesian framework all parameters are modeled, the same is the case for missing data.

The stochastic parameters in Parts 1 and 2 of this paper are N (specifying for each patient whether they have the disease) and se and sp (specifying respectively the sensitivity and specificity of each test). These parameters can be represented as par=(N,se,sn). The other quantities, α (governing sensitivity and specificity), Ψ (prevalence), and Θ (probability that each type of patient has the disease, are conditionally deterministic), given par and the data in Table 1.

There are two aspects of missing data that it are useful to distinguish. Let M be an indicator for the fact that a particular piece of potential data is not reported, and let $x_{mis}$ be the (counterfactual) value that the missing data would have taken had it been observed. Also, let $x_{obs}$ be the observed data. M is a known fact, and hence is “data” of a sort. By contrast, $x_{mis}$ is not known, and hence is a parameter with a distribution.

The key assumption about missing data is that

(10) $$p(x_{mis}|M,x_{obs},par)=p(x_{mis}|x_{obs},par)$$

What this means is that the fact that a particular datum is missing has no influence on the value of $x_{mis}$ given $x_{obs}$ and par In Rubin's [34] terminology, this assumption is missing completely at random (MCAR). Perhaps an example in which (10) is not a reasonable assumption would be enlightening.

In Pennsylvania, there are state exams for students that are high‐stakes for the students, but also for the teachers and school administrators. There is some thought that students known to be weak are discouraged from taking these exams. To the extent that this is so, the fact of being absent from school when the exam is administered suggests that the student may be weak. Of course, strong students get sick and miss exams too, but the fact of missing the exam changes the probability distribution of how well that student would have done on the exam not taken (See [35]).

Whether (10) is a reasonable assumption in a particular medical testing situation depends on why the data are not available. For example, if the design of a multicenter clinical trial of several tests specifies which tests each center is to use, then the fact that other tests have missing data is a consequence solely of the design. In this context, (10) is quite reasonable. However, if a center fails to collect data on a specific test the design calls for them to use (10) might not be appropriate, and the analysis might need to model the missing data differently. In the example analysis of missing data discussed below, (10) is assumed.

There are at least two ways of thinking about how a missing data scenario could be generated. The simpler way is to generate a single missing data scenario held constant during the analysis. A more complicated method would change $x_{mis}$ at each Gibbs step. The simpler method is more like what a data analyst would face, and so, that method is explored below. Under this scenario, $x_{obs}$ is constant over iterations.

Adaptation of the algorithm in Part 1 to the new circumstance of missing data requires a new preliminary step (step I) of imputation. In consonance with the model specified in Part 1, groups of tests are taken to be conditionally independent. The parameter α is updated with $x_{obs}$ within each group. Then (10) can be used to sample $x_{mis}$ within each group with the relevant distribution: beta‐binomial if the group has only one test, and Dirichlet multinomial otherwise.

The result of imputation is the availability of simulated complete data, so steps A, B, C and D can be implemented. However, because the “data” themselves are stochastic, programming each step can require some care.

## 4 Part IV: Missing Data, Chlamydia

### 4.1 Generation of Which Data are to be Considered Missing

To simulate which data elements are to be regarded as missing, I created a vector of length 14 204 (= 3551 × 4) of each test on each patient. Each such patient‐test combination was then given an independent 10% chance of being considered missing. Then the resulting vector was reformed into a matrix. This matrix was then counted, with the results recorded in Tables 4 and 5, divided by group.

**TABLE 4:** Counts of observed and missing for groups 1 and 2.

Status		
1	371	344
0	2798	2864
Missing	382	343
	Group 1 (DNAP)	Group 2 (culture)

**TABLE 5:** Counts of observed and missing for group 3, LCR and PCR.

		PCR		
		1	0	Missing
LCR	1	235	18	25
	0	65	2549	300
	Missing	23	305	31

These tables give the $x_{obs}$ counts for the imputations in Step I and do not change from iteration to iteration.

### 4.2 Step I: Imputation

In Group 1 (DNAP), there are from Table 4 382 missing data items, 371 patients who tested positive, and 2798 who tested negative. Then each of the 382 missing test results are imputed with a beta‐binomial draw with parameters (371+1,2798+1). Similarly, in Group 2 (culture), the 343 missing data are imputed with a beta‐binomial draw with parameters (344+1,2864+1).

The results for Group 3 (LCR and PCR) are a bit more intricate. The person‐test pairs inside the upper left 2×2 sub‐table in Table 5 are complete and do not require imputation. The remaining 5 divide into two kinds, those with one test result missing, and those with two. Partitioning a Dirichlet‐multinomial distribution yields independent Dirichlet‐multinomial pieces with the conformably partitioned hyperparameter ([36], example 2). Applied to the first kind above, yields, for missing PCR, when the LCR test is positive (i.e., = 1), a beta‐binomial with parameters (235+1,18+1). Similarly, when the LCR test is negative (i.e = 0) the missing PCR is drawn from a beta‐binomial with parameters (65 + 1, 2549). Symmetrically, when the LCR test is missing, it is drawn from a beta‐binomial (235+1,65+1) when PCR is positive (= 1), and beta‐binomial (18+1,2549+1) when PCR is negative (=0). Finally, the second kind above is a draw from a Dirichlet multinomial with parameter (235+1,18+1,65+1,2549+1) respectively yielding results {(1,1),(1,0),(0,1)and(0,0)}.

After imputations, the result (for this iteration) is a full data set with no missing test results. Hence, steps A, B, C and D (in that order) can be executed, returning to step I to choose new imputations for the missing values.

### 4.3 Algorithm Implemented

Again, I report on a run of 10 000 iterations. Because of the added uncertainty caused by the missing data, I chose a burn‐in of 1000. Again, the starting vector for θ was 0.5 repeated 16 times.

The Geweke tests for convergence are reported in Table 6.

**TABLE 6:** Geweke convergence test results, missing data case.

DNAP	Culture	LCR	PCR
Sensitivity			
−1.077	−0.432	1.159	0.445
Specificity			
−0.571	−1.413	0.430	0.410

**FIGURE 5:**
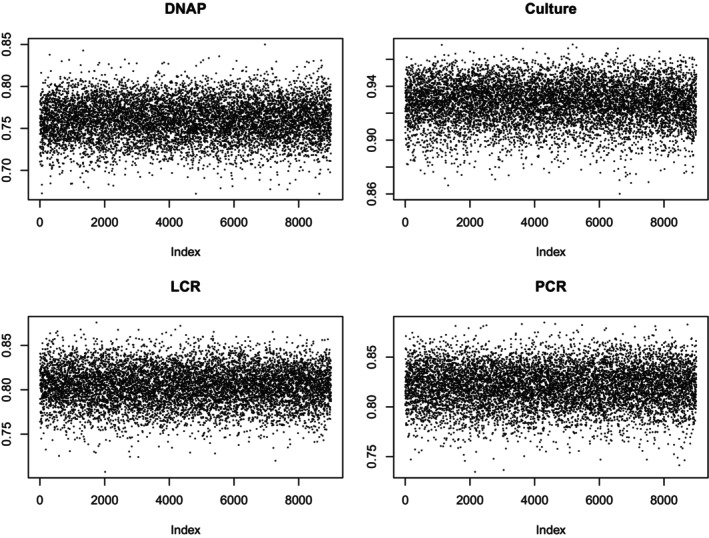
Sensitivity Trace Plots, Missing Data.

**FIGURE 6:**
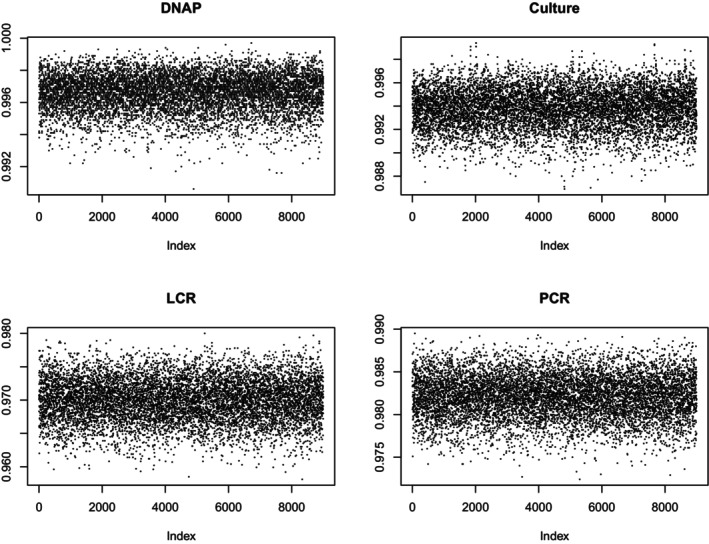
Specificity Trace Plots, Missing Data.

Again, these tests suggest satisfactory convergence. Figures 5 and 6 are trace plots for the sensitivity and specificity of the tests. They show no trends, so I accept that the sampler has converged.

**FIGURE 7:**
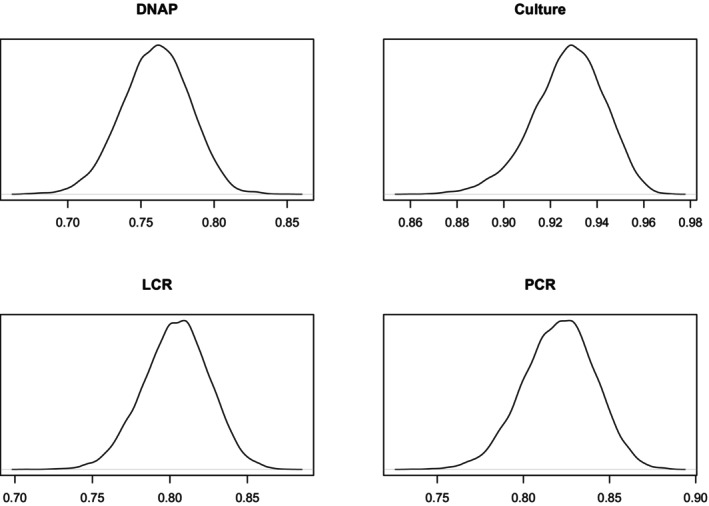
Sensitivity Density Plots, Missing Data.

**FIGURE 8:**
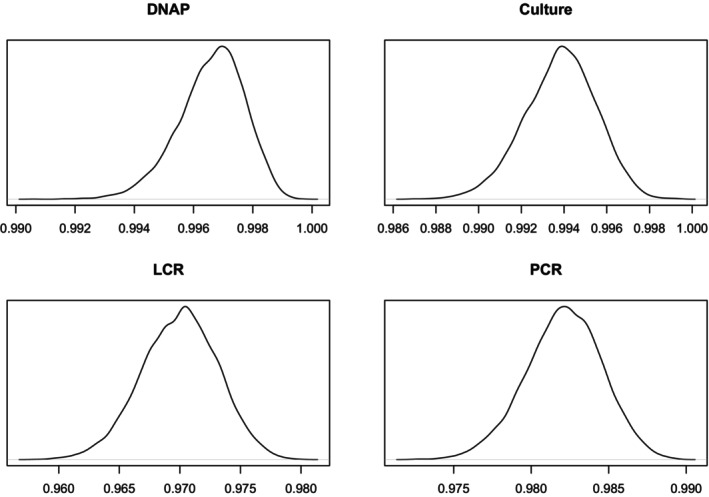
Specificity Density Plots, Missing Data.

Table 7 shows a comparison of the results with missing data to those without missing data. The results are broadly similar, although the LCR sensitivity medians with missing data is outside the 95% credible intervals calculated on the full data set. Figures 7 and 8 display the density plots for specificities and sensitivities, respectively.

**TABLE 7:** Comparison of missing data results to results with no missing data, median and 95% credible intervals.

		Missing data results	No missing data results
Specificity	DNAP	0.9967 (0.9941, 0.9985)	0.9966 (0.9940, 0.9983)
	Culture	0.9939 (0.9903, 0.9969)	0.9933 (0.9897, 0.9962)
	LCR	0.9701 (0.9635, 0.9759)	0.9691 (0.9631, 0.9752)
	PR	0.9822 (0.9970, 0.9867)	0.9731 (0.9670, 0.9785)
			
Sensitivity	DNAP	0.7607 (0.7134, 08037)	0.8055 (0.7596, 0.8475)
	Culture	0.9286 (0.8942, 0.9546)	0.9538 (0.9233, 0.9748)
	LCR	0.8046 (0.7606, 0.8433)	0.8688 (0.8282, 0.9032)
	PCR	0.8213 (0.7787, 0.8590)	0.8127 (0.7673, 0.8524)

Again, Table A2 in the Appendix gives more detail on the results.

## 5 Part V: Concluding Discussion

This paper proposes a Dirichlet multinomial to analyze the results of several binary tests given simultaneously. It shows that the computations are feasible. The assumptions are qualitative in nature: it divides the tests into independent groups, and within groups allows for arbitrary dependence. A disadvantage of this model is that a group of size g introduces $2^{g}$ parameters, which gets sizable very quickly as a function of g.

The application to tests of chlamydia shows that all four tests have very high specificity, and that culture has a substantially higher sensitivity than do the other tests.

Oddly enough, the issue of lack of identification did not hinder the Gibbs sampler in the application. The thought that the “real” parameter space is folded upsets the kinds of metrics used to monitor convergence of these samplers. An examination of the full implications of the approach to the lack of identification remains an open issue.

## Funding

The author has nothing to report.

## Conflicts of Interest

The author declares no conflicts of interest.

## Supporting information

Data S1: sim70372‐sup‐0001‐Supinfo.rtf.

## Data Availability

The data that support the findings of this study are available in PubMed at https://pubmed.ncbi.nlm.nih.gov/19067379/, reference number 19067379. These data were derived from the following resources available in the public domain:—Statistics in Medicine, https://onlinelibrary.wiley.com/doi/10.1002/sim.3470.

## Appendix A

**TABLE A1:** Specificity and sensitivity results, complete data.

	Summary results: Complete data						
		Min	1st Q	Median	Mean	3rd Q	Max
Specificity	DNAP	0.9906	0.9958	0.9966	0.9965	0.9973	0.9997
	Culture	0.9857	0.9922	0.9933	0.9932	0.9944	0.9982
	LCR	0.9573	0.9673	0.9694	0.9694	0.9715	0.9794
	PCR	0.9585	0.9711	0.9731	0.9730	0.9750	0.9827
							
Sensitivity	DNAP	0.6970	0.7897	0.8055	0.8049	0.8208	0.8871
	Culture	0.8722	0.9445	0.95325	0.9524	0.9618	0.9884
	LCR	0.7875	0.8557	0.8688	0.8680	0.8815	0.9280
	PCR	0.7277	0.7975	0.8127	0.8119	0.8269	0.8817

**TABLE A2:** Specificity and sensitivity results, incomplete data.

	Summary results: Incomplete data						
		Min	1st Q	Median	Mean	3rd Q	Max
Specificity	DNAP	0.9906	0.9959	0.9867	0.9966	0.9974	0.9997
	Culture	0.9869	0.9927	0.9939	0.9938	0.9940	0.9994
	LCR	0.9581	0.9678	0.9701	0.9700	0.9721	0.9800
	PCR	0.9724	0.9805	0.9822	0.9821	0.9838	0.9895
							
Sensitivity	DNAP	0.6720	0.7450	0.7607	0.7602	0.7760	0.8500
	Culture	0.8601	0.9181	0.9286	0.9277	0.9384	0.9711
	LCR	0.7075	0.7899	0.8046	0.8040	0.8185	0.8759
	PCR	0.7346	0.8070	0.8213	0.8206	0.8348	0.8849
