# Supplementary material for: A Dirichlet‐Multinomial Gibbs Algorithm for Assessing the Accuracy of Binary Tests in the Absence of a Gold Standard
Source: Stat Med. 2026 Jan 22;45(1-2):e70372. doi: 10.1002/sim.70372 (PMC12828250; doi:10.1002/sim.70372)
Supplement: Supplementary file 1 — Data S1: sim70372‐sup‐0001‐Supinfo.rtf. [file SIM-45-0-s001.rtf]

 All programs are in Base r. I used Rstudio.  I have divided the programs into two sections, those relevant to PartII of the paper (no missing data) and those relevant to Part IV (missingdata).     FOR PART II    READMEDat1 is Table 1 in the paper, excluding the two rows with no data.function(x){     # FOR USE IN THE NO MISSING DATA BASE CASE#The basic functions to use are gibbs1 and anal1.the calls are:#  gibbs1(c(10000,start)) to run a chain of length 10000 using as the first# omega vector "start ", of length 14. The output is a matrix of size# 10000 x 8, giving the sensitivity and speciifcity of each test. You might# want to end this run this with summary(gibbs(c(10000,start))) toshorten the output.# anal1(10000) produces summary, Geweke tests and figures of the output.#the flow is anal1 calls gibbs1, which calls calc1. calc1 does thecalculations for a single# iteration, gibbs1 implements the gibbs sampler over many iterations,and anal1 manages the# output, including the graphs# calc1 calls rdirichlet.}      CALC1calc1<-     function(y){     # this function takes a single input theta, and produces an outputof (results,(new)theta)     theta=y[1:14]     posi=rep(NA,14)     negi=rep(NA,14)     results=rep(NA,8)     for (j in 1:14){         posi[j]=rbinom(1,dat1$obs[j],theta[j])        negi[j]=dat1$obs[j]-posi[j]         # this yields vectors of length 14 of the positive and negative         # simulated results on who has the condition     }     #sen3 [only cases patient is positive !]     test3right=sum((dat1$test3[])*posi[])     test3wrong=sum((1-dat1$test3[])*posi[])     sen3=rbeta(1,test3right+1,test3wrong+1)     results[1]=sen3     #spec3 [only cases where patient is negative ]     test3right=sum((1-dat1$test3[])*negi[])     test3wrong=sum((dat1$test3[])*negi[])     spec3=rbeta(1,test3right+1,test3wrong+1)     results[2]=spec3     #sen4     test4right=sum((dat1$test4[])*posi[])     test4wrong=sum((1-dat1$test4[])*posi[])     sen4=rbeta(1,test4right+1,test4wrong+1)     results[3]=sen4     #spec4     test4right=sum((1-dat1$test4[])*negi[])     test4wrong=sum((dat1$test4[])*negi[])     spec4=rbeta(1,test4right+1,test4wrong+1)     results[4]=spec4     #sen12     s11=sum(posi[1:4])     s10=sum(posi[5:7])     s01=sum(posi[8:10])     s00=sum(posi[11:14])     sen12=rdirichlet(c(s11+1,s10+1,s01+1,s00+1))     results[5]=sen12[1]+sen12[2]     results[6]=sen12[1]+sen12[3]     #spec12     t11=sum(negi[1:4])     t10=sum(negi[5:7])     t01=sum(negi[8:10])     t00=sum(negi[11:14])     spec12=rdirichlet(c(t11+1,t10+1,t01+1,t00+1))     results[7]=spec12[4]+spec12[3]     results[8]=spec12[4]+spec12[2]     # next comes prevalence     psi=sum(dat1$obs[]*theta[])/sum(dat1$obs[])     # Bayes Theoremf11=psi*sen12[1]*(sen3**(dat1$test3[1:4]))*((1-sen3)**(1-dat1$test3[1:4]))*(sen4**dat1$test4[1:4])*((1-sen4)**(1-dat1$test4[1:4]))f21=(1-psi)*spec12[1]*(spec3**(1-dat1$test3[1:4]))*((1-spec3)**(dat1$test3[1:4]))*(spec4**(1-dat1$test4[1:4]))*((1-spec4)**(dat1$test4[1:4]))     g1=f11/(f11+f21)f12=psi*sen12[2]*(sen3**(dat1$test3[5:7]))*((1-sen3)**(1-dat1$test3[5:7]))*(sen4**dat1$test4[5:7])*((1-sen4)**(1-dat1$test4[5:7]))f22=(1-psi)*spec12[2]*(spec3**(1-dat1$test3[5:7]))*((1-spec3)**(dat1$test3[5:7]))*((1-spec4)**dat1$test4[5:7])*(spec4**(1-dat1$test4[5:7]))      g2=f12/(f12+f22)f13=psi*sen12[3]*(sen3**(dat1$test3[8:10]))*((1-sen3)**(1-dat1$test3[8:10]))*(sen4**dat1$test4[8:10])*((1-sen4)**(1-dat1$test4[8:10]))f23=(1-psi)*spec12[3]*(spec3**(1-dat1$test3[8:10]))*((1-spec3)**(dat1$test3[8:10]))*((1-spec4)**dat1$test4[8:10])*(spec4**(1-dat1$test4[8:10]))     g3=f13/(f13+f23)f14=psi*sen12[4]*(sen3**dat1$test3[11:14])*((1-sen3)**(1-dat1$test3[11:14]))*(sen4**dat1$test4[11:14])*((1-sen4)**(1-dat1$test4[11:14]))f24=(1-psi)*spec12[4]*(spec3**(1-dat1$test3[11:14]))*((1-spec3)**(dat1$test3[11:14]))*(spec4**(1-dat1$test4[11:14]))*((1-spec4)**(dat1$test4[11:14]))     g4=f14/(f14+f24)     theta=c(g1,g2,g3,g4)     return(c(results,theta))      }RDIRICHLETfunction (alpha){     l=length(alpha)     x=rgamma(l,alpha)     y=x/sum(x)     return(y)}GIBBS1  function(x){     # input: its,theta (length 14)     # calls calc1; returns matrix:its x 8 of test sensitivities andspecificities     its=x[1]     theta=x[2:15]     m=rep(NA,22)     out=matrix(NA,nrow=its,ncol=8)colnames(out)=c("senDNAP","spDNAP","senCulture","spCulture","senLCR","senPCR","spLCR","spPCR")# setting the seed allows one to rerun the sampler and get the same output     set.seed(478)     m=calc1(theta)     out[1,]=m[1:8]     theta=m[9:22]     # this loop performs the Gibbs sampling     for (i in 2:its){     m=calc1(theta)     out[i,]=m[1:8]     # this updates theta     theta=m[9:22]     }     out=round(out,digits=4)    return(out)}ANAL1function(n){     #started Feb.1,2025     #  automates analysislibrary(coda)x=matrix(NA, nrow=6,ncol=8)s=mcmc(gibbs(c(n,rep(0.5,14))))#note burn-in set at 1000 in next liness=s[1000:n,]zq=c("senDNAP","spDNAP","senCulture","spCulture","senLCR","senPCR","spLCR","spPCR")t=matrix(NA,nrow=8,ncol=2)rownames(t)=zqcolnames(t)=c("q 0.025","q 0.975")for (i in 1:8){t[i,]=quantile(ss[,i],probs=(c(0.025,0.975)))}show(t)w=geweke.diag(ss,frac1=0.1,frac2=0.5)show(w)par(mfrow=c(2,2))plot(ss[,2],main="DNAP",ylab="",cex=0.1)plot(ss[,4],main="Culture",ylab="",cex=0.1)plot(ss[,5],main="LCR",ylab="",cex=0.1)plot(ss[,6],main="PCR",ylab="",cex=0.1)mtext("SPECIFICITY TRACE PLOTS",side=3,outer=TRUE,line=-1.5)dev.copy2pdf(file="~kadane/Documents/spctrace16.pdf")dev.off()par(mfrow=c(2,2))plot(ss[,1],main="DNAP",ylab="",cex=0.1)plot(ss[,3],main="Culture",ylab="",cex=0.1)plot(ss[,7],main="LCR",ylab="",cex=0.1)plot(ss[,8],main="PCR",ylab="",cex=0.1)mtext("SENSITIVITY TRACE PLOTS",side=3,outer=TRUE,line=-1.5)dev.copy2pdf(file="~kadane/Documents/sentrace16.pdf")dev.off()par(mfrow=c(2,2))plot(density(ss[,2],adjust=1),main="DNAP",ylab="",xlab="",yaxt="n")plot(density(ss[,4],adjust=1),main="Culture",ylab="",xlab="",yaxt="n")plot(density(ss[,5],adjust=1),main="LCR",ylab="",xlab="",yaxt="n")plot(density(ss[,6],adjust=1),main="PCR",ylab="",xlab="",yaxt="n")mtext("SPECIFICITY DENSITY PLOTS",side=3,outer=TRUE,line=-1.5)dev.copy2pdf(file="~kadane/Documents/spcdensity16.pdf")dev.off()par(mfrow=c(2,2))plot(density(ss[,1],adjust=1),main="DNAP",ylab="",xlab="",yaxt="n")plot(density(ss[,3],adjust=1),main="Culture",ylab="",xlab="",yaxt="n")plot(density(ss[,7],adjust=1),main="LCR",ylab="",xlab="",yaxt="n")plot(density(ss[,8],adjust=1),main="PCR",ylab="",xlab="",yaxt="n")mtext("SENSITIVITY DENSITY PLOTS",side=3,outer=TRUE,line=-1.5)dev.copy2pdf(file="~kadane/Documents/sendensity16.pdf")dev.off()x=summary(ss)show(x)return(w)}  FOR PART IVREADMEPRELIMSdat is Table 1 in the paper with 16 rowsfunction(x){     # THIS IS FOR THE MISSING DATA CASE# This records the steps taken to make Table 4, useful# for the computations that follow.# 1. The matrix "dat" is strung out in a vector#    of Length 14204=3551 x 4, using function xq#    z=rep(NULL,14204)#    z=rep(getv(1),dat[1,5])  #   for (j in 2:16){   #      z=c(z,rep(getv(j),dat[j,5]))}#    return(z)# The results of xq are stored in datstr#    2. datstr is now sampled to decide which data locations are# to be regarded as missing. The command is# hb=   sample(datstr,size=14204,prob=rep(0.1,14204) )# hb is then multiplied by 2.# 3. ready=pmax(hb,datstr)     # Hence "ready" is a string of 0's, 1's and 2"s in which a 0 means that     # the patient is tested negatve, 1 that the patient is testedpositive, and     # 2 the data is now to be regarded as missing.#4. ready is now reformed into a matrix:  #   readymtrx=matrix(ready,nrow=3551,ncol=4,byrow=TRUE).#5. Now "table" is applied to columns 1 and 2 of readymtrx, yieldresults recorded in     # Table 4 of the paper for DNAP and Culture, respectively.#6. Columns 3 and 4 require special handling because they jointlyconstitute a group.     # I created score=readymtrx[,3]+(10*readymtrx[,4])     # applying table to score yields the rest of the results in Table 4.# CHECKED APRIL 19.}CALCfunction(y){     # this function takes a single input oldtheta, and produces anoutput of (results,theta)     theta=y           # IMPUTATION  # April 6,2025  impute for missing data in readymtrx  # the next lines impute for DNAP and Culture, respectively     for (j in 1:3551){         if (readymtrx[j,1]==2){readymtrx[j,1]<- rbetabinom(1+371,1+2798)}         if (readymtrx[j,2]==2){readymtrx[j,2]<- rbetabinom(1+344,1+2864)}     }    # Now for LCR and PCR     for (j in 1:3551){         # the next lines handle cases in which only one of test3 andtest4 are missing         if ((readymtrx[j,3]==2 ) &(readymtrx[j,4]==1)){readymtrx[j,3]<-rbetabinom(235+1,65+1)}         if ((readymtrx[j,3]==2 ) &(readymtrx[j,4]==0)){readymtrx[j,3]<-rbetabinom(18+1,2549+1)}         if ((readymtrx[j,4]==2 ) &(readymtrx[j,3]==1)){readymtrx[j,4]<-rbetabinom(235+1,18+1)}         if ((readymtrx[j,4]==2 ) &(readymtrx[j,3]==0)){readymtrx[j,4]<-rbetabinom(65+1,2549+1)}         # these lines handle cases in which both tests3 and 4 are missing         vd=matrix(NA,nrow=3551,ncol=4)         vd[j,]=rmultinom(1,1,rdirichlet(c(235,18,65,2549)))         #now set up the ifs         if((readymtrx[j,3]==2) & (readymtrx[j,4]==2)){             if(vd[j,1]==1){(readymtrx[j,3]=1)}              if (vd[j,1]==1) {readymtrx[j,4]=1}             if (vd[j,2]==1) {(readymtrx[j,3]=1)}              if (vd[j,2]==1){   (readymtrx[j,4]=0)}             if (vd[j,3]==1) {(readymtrx[j,3]=0)}              if(vd[j,3]==1)  { (readymtrx[j,4]=1)}             if (vd[j,4]==1){(readymtrx[j,4]=0)}              if (vd[j,4]==1){readymtrx[j,3]=0}             } #closes main if 9 lines above     }   #closes j loop         #  SAMPLING DISEASE STATUS     zt=rep(NA,3551)     zr=rep(NA,3551)     for (j in 1:3551){         zt[j]=makev1(readymtrx[j,1:4])         zr[j]=theta[zt[j]]#        readymtrx[j,5]<-zr[j]         readymtrx[j,5]<-rbinom(n=1,prob=zr[j],size=1)     }     # this yields vectors  of the positive and negative     # simulated results on who has the condition              # DERIVING SENSITIVITY AND SPECIFICITY     #sen3 [only cases patient is positive !]     test3right<-sum((readymtrx[,3]==1)*(readymtrx[,5]==1))     test3wrong<-sum((readymtrx[,3]==0)*(readymtrx[,5]==1))     sen3=rbeta(1,test3right+1,test3wrong+1)     results[1]<-sen3     #spec3 [only cases where patient is negative ]     test3right<-sum((readymtrx[,3]==0)*(readymtrx[,5]==0))     test3wrong<-sum((readymtrx[,3]==1)*(readymtrx[,5]==0))     spec3<-rbeta(1,test3right+1,test3wrong+1)     results[2]<-spec3     #sen4     test4right<-sum((readymtrx[,4]==1)*(readymtrx[,5]==1))     test4wrong<-sum((readymtrx[,4]==0)*(readymtrx[,5]==1))     sen4=rbeta(1,test4right+1,test4wrong+1)     results[3]=sen4     #spec4     test4right<-sum((readymtrx[,4]==0)*(readymtrx[,5]==0))     test4wrong<-sum((readymtrx[,4]==1)*(readymtrx[,5]==0))     spec4=rbeta(1,test4right+1,test4wrong+1)     results[4]=spec4     #sen12s11=sum((readymtrx[,1]==1)*(readymtrx[,2]==1)*(readymtrx[,5]==1))s10<-sum((readymtrx[,1]==1)*(readymtrx[,2]==0)*(readymtrx[,5]==1))s01<-sum((readymtrx[,1]==0)*(readymtrx[,2]==1)*(readymtrx[,5]==1))s00<-sum((readymtrx[,1]==0)*(readymtrx[,2]==0)*(readymtrx[,5]==1))     sen12=rdirichlet(c(s11+1,s10+1,s01+1,s00+1))     results[5]=sen12[1]+sen12[2]     results[6]=sen12[1]+sen12[3]     #spec12t11=sum((readymtrx[,1]==1)*(readymtrx[,2]==1)*(readymtrx[,5]==0))t10=sum((readymtrx[,1]==1)*(readymtrx[,2]==0)*(readymtrx[,5]==0))t01=sum((readymtrx[,1]==0)*(readymtrx[,2]==1)*(readymtrx[,5]==0))t00=sum((readymtrx[,1]==0)*(readymtrx[,2]==0)*(readymtrx[,5]==0))     spec12=rdirichlet(c(t11+1,t10+1,t01+1,t00+1))     results[7]=spec12[4]+spec12[3]     results[8]=spec12[4]+spec12[2]        # DERIVING PREVALENCE     psi=(sum(readymtrx[,5])+1)/(3551+2)#    psi=1-psi     # NEW THETA USING BAYES THEOREM     ff=rep(NA,16)     factor11=rep(NA,16)     factor12<-rep(NA,16)     factor13=rep(NA,16)     for (j in 1:16){         if (getv(j)[1]==1) {             factor11[j]=sen3         } else factor11[j]=(1-sen3)         if (getv(j)[2]==1) {             factor12[j]=sen4         } else factor12[j]=(1-sen4)         if (getv(j)[3]==1 & getv(j)[4]==1){             factor13[j]=sen12[1]         } else if (getv(j)[3]==1 & getv(j)[4]==0){             factor13[j]=sen12[2]         } else if (getv(j)[3]==0 & getv(j)[4]==1){             factor13[j]=sen12[3]         } else factor13[j]=sen12[4]         ff[j]=psi*factor11[j]*factor12[j]*factor13[j]}    gg=rep(NA,16)    factor21=rep(NA,16)    factor22=rep(NA,16)    factor23=rep(NA,16)     for (j in 1:16){         if (getv(j)[1]==0) {             factor21[j]=(spec3)         } else factor21[j]=(1-spec3)         if (getv(j)[2]==0) {             factor22[j]=(spec4)         } else factor22[j]=(1-spec4)         if (getv(j)[3]==0 & getv(j)[4]==0){             factor23[j]=(spec12[1])         } else if (getv(j)[3]==0 & getv(j)[4]==1){             factor23[j]=(spec12[2])         } else if (getv(j)[3]==1 & getv(j)[4]==0){             factor23[j]=(spec12[3])         } else factor23[j]=(spec12[4])         gg[j]=(1-psi)*factor21[j]*factor22[j]*factor23[j] }     theta=ff/(ff+gg)    return(c(results,theta))}xqfunction(x){     z=rep(NULL,14204)     z=rep(getv(1),dat[1,5])     for (j in 2:16){       z=c(z,rep(getv(j),dat[j,5]))     }     return(z)}getvfunction(x){# this function finds the  vector of test results x#related to the line # in dat     M=length(x)     ans=rep(NULL,4*M)     w=rep(NA,4)     x=16-x     for (i in 1:M){     w[1]=x[i]%/%8     w[2]=(x[i]-w[1]*8)%/%4     w[3]=(x[i]-w[1]*8-w[2]*4)%/%2     w[4]=x[i]-w[1]*8-(w[2]*4)-(w[3]*2)     ans=c(ans,w)     }     ans=ans[]     return(ans)}rbetabinomfunction(s1,s2){   y<-rbinom(1,1,prob=rbeta(1,s1,s2))   return(y)}makevfunction(x){# x is a vector of length 4L of 0's and 1"s# this function returns the lines in dat related to the#    vector of test results     L=length(x)/4     z=rep(NA,L)     for (i in 1:L){         w=c(x[i*4-3],x[i*4-2],x[i*4-1],x[i*4])     z[i]=16-(8*w[1])-(4*w[2])-(2*w[3])-w[4]     }     return(z)     }GIBBS  function(x){     # input: its, theta(length16) for missing data case     # calls  calc     #returns matrix:its x 8 of test sensitivities and specificities     its=x[1]     theta=rep(NA,16)     theta=x[2:17]     m=matrix(NA,nrow=its,ncol=24)     mx=rep(NA,24)     out=matrix(NA,nrow=its,ncol=8)  #  colnames(out)=c("senDNAP","spDNAP","senCulture","spCulture","senLCR","senPCR","spLCR","spPCR")# setting the seed allows one to rerun the sampler and get the same output     set.seed(478)     mx=calc(theta)     out[1,1:8]=mx[1:8]     theta[1:16]=mx[9:24]     # this loop performs the Gibbs sampling     for (i in 2:its){     m[i,]=calc(theta)     out[i,]=m[i,1:8]     # this updates theta     theta=m[i,9:24]}      return(out)}ANAL:function(n){     #started Feb.1,2025     #  automates analysislibrary(coda)x=matrix(NA, nrow=6,ncol=8)s=mcmc(gibbs(c(n,rep(0.5,16))))#note burn-in set at 1000 in next liness=s[1000:n,]zq=c("senDNAP","spDNAP","senCulture","spCulture","senLCR","senPCR","spLCR","spPCR")t=matrix(NA,nrow=8,ncol=2)rownames(t)=zqcolnames(t)=c("q 0.025","q 0.975")for (i in 1:8){t[i,]=quantile(ss[,i],probs=(c(0.025,0.975)))}show(t)w=geweke.diag(ss,frac1=0.1,frac2=0.5)par(mfrow=c(2,2))plot(ss[,1],main="DNAP",ylab="",cex=0.1)plot(ss[,3],main="Culture",ylab="",cex=0.1)plot(ss[,7],main="LCR",ylab="",cex=0.1)plot(ss[,8],main="PCR",ylab="",cex=0.1)mtext("SENSITIVITY TRACE PLOTS,MISSING DATA",side=3,outer=TRUE,line=-1.5)pdf(file="~kadane/Documents/sentrace115.pdf")#dev.copy2pdfdev.off()par(mfrow=c(2,2))plot(ss[,2],main="DNAP",ylab="",cex=0.1)plot(ss[,4],main="Culture",ylab="",cex=0.1)plot(ss[,5],main="LCR",ylab="",cex=0.1)plot(ss[,6],main="PCR",ylab="",cex=0.1)mtext("SPECIFICITY TRACE PLOTS,MISSING DATA",side=3,outer=TRUE,line=-1.5)pdf(file="~kadane/Documents/spctrace115.pdf")dev.off()par(mfrow=c(2,2))plot(density(ss[,2],adjust=1),main="DNAP",ylab="",xlab="",yaxt="n")plot(density(ss[,4],adjust=1),main="Culture",ylab="",xlab="",yaxt="n")plot(density(ss[,5],adjust=1),main="LCR",ylab="",xlab="",yaxt="n")plot(density(ss[,6],adjust=1),main="PCR",ylab="",xlab="",yaxt="n")mtext("SPECIFICITY DENSITY PLOTS,MISSING DATA",side=3,outer=TRUE,line=-1.5)pdf(file="~kadane/Documents/spc3density115.pdf")dev.off()par(mfrow=c(2,2))plot(density(ss[,1],adjust=1),main="DNAP",ylab="",xlab="",yaxt="n")plot(density(ss[,3],adjust=1),main="Culture",ylab="",xlab="",yaxt="n")plot(density(ss[,7],adjust=1),main="LCR",ylab="",xlab="",yaxt="n")plot(density(ss[,8],adjust=1),main="PCR",ylab="",xlab="",yaxt="n")mtext("SENSITIVITY DENSITY PLOTS,MISSING DATA",side=3,outer=TRUE,line=-1.5)pdf(file="~kadane/Documents/sen3density115.pdf")dev.off()show(summary(ss))return(w)}
